# Noncommunicable disease communication campaigns in the Pacific Region: strengths, challenges and lessons learned from an online survey and poster analysis

**DOI:** 10.5365/wpsar.2025.16.4.1234

**Published:** 2025-12-17

**Authors:** Ferdinand Strobel, Solène Bertrand Protat

**Affiliations:** a Public Health Division, The Pacific Community, Nouméa, New Caledonia.

## Abstract

**Objective:**

Noncommunicable diseases impose a significant and growing burden in Pacific island countries and territories, prompting health authorities to implement media-driven communication campaigns as part of their broader strategies to address these diseases and promote health. This analysis was undertaken to assess the strengths and limitations of these campaigns and identify areas for improvement.

**Methods:**

A semi-structured online survey, conducted between August and October 2023, and a visual content analysis were used to examine noncommunicable disease-related communication in Pacific island countries and territories. Distributed through the Pacific Community’s Public Health Division focal-point network, the survey was designed to gather qualitative insights on campaign development, evaluation, audience targeting, challenges and success factors. Public health posters from the Pacific Community’s archives were analysed using a semiological approach to categorize their enunciative functions.

**Results:**

Thirty-four survey responses from 12 Pacific island countries and territories were received. Tobacco was the top campaign issue, followed by nutrition, physical activity and cancer awareness; social media was the primary communication channel. Most respondents (80%) rated their campaigns as only moderately successful. Actions needed to enhance the impact of communications included better planning, more preliminary research, sustainable funding, skilled staff and greater cross-sector collaboration. Most noncommunicable disease-related posters (70%) served a representational function by portraying reality as designed by public health authorities. While most posters incorporated local cultural elements and vernacular languages, they were predominantly authoritative (46%) or neutral (44%) in tone. Furthermore, 73% were not tailored to specific target groups.

**Discussion:**

Enhancing communication requires greater resourcing, transdisciplinary approaches and stronger audience engagement. More research-informed strategies that integrate behavioural science could improve interventions that promote healthier choices. Achieving this necessitates closer collaboration across disciplines, and stronger partnerships and engagement with communities.

Noncommunicable diseases (NCDs) such as cardiovascular diseases, cancers, diabetes and chronic respiratory diseases are the leading cause of premature death in Pacific island countries and territories (PICTs). (*1*) More than 80% of adults are overweight or obese, 33% have elevated blood pressure and 40% exhibit high cholesterol. (*2*) Diabetes prevalence is among the highest globally, with rates exceeding 20% in several nations. (*3*) The burden of NCDs on families, health-care systems and national economies is such that PICT leaders have described the situation as a “human, social and economic crisis” and a threat to sustainable human development. (*4*)

PICTs are responding by improving health-care service delivery for screening, management and care. (*5*) Many are also adopting population-level policy measures recommended by the World Health Organization (WHO) to regulate tobacco and alcohol, and to promote access to nutritious foods and physical activity. (*6*) However, many health systems remain overwhelmed and underresourced, and regulatory measures around tobacco control, alcohol and food are often inconsistently implemented and poorly enforced. (*7*)

As part of their response to the public health threat posed by NCDs, many PICTs have employed communication campaigns to promote healthier lifestyles. Communication can shape public understanding of NCD risk factors and influence health behaviours. However, to be effective, communication must be culturally sensitive, linguistically appropriate and grounded in local context. The use of multiplatform approaches (for example, mass media, social media, posters, interpersonal communication) can increase message reach and reinforce impact. Evidence suggests that well designed communication can help shift social norms and influence policy changes towards healthier lifestyles. (*8*, *9*)

Several PICTs have implemented media-driven communication initiatives as part of their NCD prevention strategies. Distinct from direct, interpersonal or individual forms of communication, media-driven initiatives are influenced and facilitated by various forms of public media including television, radio, newspaper, and the public display of banners and posters. However, thus far, the evaluation of the effectiveness of NCD-related communication in PICTs has been limited.

The aim of this study, which has been ongoing since 2017, was to inform a Pacific Community (SPC) capacity-building project on NCD prevention communication. Its objectives were to generate insights from practitioners’ lived experiences of NCD prevention and to produce actionable recommendations to enhance the design and implementation of future communication efforts. We conducted an online survey as the primary method of data collection and complemented it with a poster analysis as an additional source of evidence.

## Methods

We conducted a semi-structured questionnaire-based online survey using Google Forms, which was distributed to all 22 PICTs through SPC’s Public Health Division (PHD) NCD focal-point network. The NCD focal points are officials of health ministries (or departments) who are mandated to liaise with the PHD on all matters related to NCDs. These individuals were contacted by e-mail and asked to pass on the survey link to relevant personnel within their ministries or affiliated entities (for example, health promotion units) that are directly responsible for or involved in NCD prevention communication and health promotion. Participation was voluntary and anonymous; only the identity of participating country or territory was recorded. The independent principal researcher (FS) was the only person who had sight of respondents’ e-mail addresses, ensuring that all information was kept confidential.

The questionnaire was specifically created for this study, as there was no existing standardized relevant questionnaire that suited this research. Moreover, since this research was primarily intended to inform a SPC capacity-building project, the design of the questionnaire was guided by the needs of the project rather than the need to ensure comparability with other studies. Our questionnaire design approach was theoretically grounded in the Socio-ecological Model, (*10*) which enabled us to explore not only message design, but also institutional constraints, community dynamics and broader system-level communication challenges.

The questionnaire comprised 13 questions – eight open-ended and five closed – and aimed to gather qualitative data on campaign development, evaluation, themes, audience targeting, perceptions of success, success factors and challenges (questionnaire available upon request). Respondents were first asked if they were directly involved in a communication campaign on NCDs. They were also invited to provide recommendations for improving NCD-related communication campaigns based on their direct experience. Prior to dissemination, the questionnaire was reviewed by SPC’s bilingual public health experts for quality assurance and translation accuracy (French and English versions).

Data analysis identified thematic areas that aligned with the questionnaire. These comprised three broad themes: campaign execution (success and challenge factors); the relevance of messaging for the target audience (specificity, messaging/content adequacy, relevance of channels, cohesiveness and consistency); and engagement for impact (public engagement, accessibility, follow-up services outcome and impact observed). The qualitative data from the questionnaires were manually reviewed and the responses were categorized by theme. Data were entered into Microsoft Excel for descriptive analysis.

**Fig. 1:**
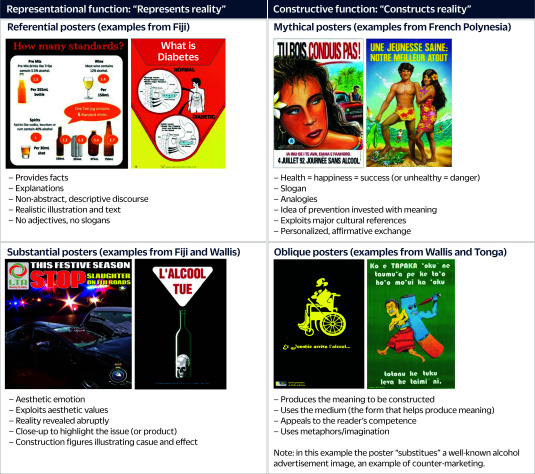
Examples of posters by enunciative functions^a^

In addition, we analysed undated public health posters that addressed NCDs. They were sourced from SPC’s PHD archives and analysed using a matrix to identify source, theme, text presence, figures, language, visual style, cultural references, tone, target and motivator. Posters were categorized by their enunciative functions using Lebel’s method for analysing public health images, (*11*) distinguishing between “representational” and “constructive” functions. Representational function includes referential and substantial compositions, while constructive function includes mythical and oblique compositions. Examples of each type of poster are shown in **Fig. 1**.

[**insert** Figure 1]

## Results

### Online survey

A total of 34 responses were received from 12 of the 22 PICTs. Of the 34 respondents, 29 (85%) were at that time directly involved in NCD prevention communication campaigns (100% in the Pacific island countries [PICs] and 75% in the territories). Respondents represented New Caledonia (*n* = 11), French Polynesia (*n* = 8), and Cook Islands, Fiji, Marshall Islands, Federated States of Micronesia, Nauru, the Commonwealth of the Northern Mariana Islands, Palau, Papua New Guinea, Samoa, the Solomon Islands, Tokelau, Tonga, Tuvalu, Vanuatu, and Wallis and Futuna (*n* = 15, one from each). The over-representation of the territories in the responses is attributed to larger resources, including staff dedicated to NCD communication in the French territories compared with PICs. Tobacco control was the top campaign issue, followed by nutrition and physical activity. Cancer awareness, especially breast cancer through campaigns like Pink October, was also prominent. Diabetes was frequently addressed due to its high prevalence. A campaign on youth screen addiction highlighted digital health issues. Integrated campaigns targeting multiple risk factors were common.

The most used channels were web sites/social media, press and radio/television (each employed by over 80% of survey respondents). Around half reported employing posters, community discussions and health-worker interventions. Least used were champions (by 38%), artistic productions and preaching in a religious setting (by less than 30%). Social and mass media were preferred for effective dissemination, but community discussions, one-to-one engagement, school programmes, champions, storytelling, arts, roadshows and workplace initiatives, although less used, were also considered important channels. The value of employing multiple channels was acknowledged.

Overall, 62% of respondents stated that messages were designed for audiences in local languages, and over half reported engaging communication professionals. Preliminary research and message testing were less common (35–38%), and evaluation and follow-up even more so (18–21%). Borrowing standard messages from other countries was a more frequent practice in PICs (64%) than in the territories (20%). Among PIC respondents, only 28% stated that campaigns included preliminary studies, compared with 45% from the territories. The use of communication professionals was also a less common practice in PICs than in the territories (29% vs 70%), a further indication of greater resource availability in the territories. Cultural aspects and beliefs were crucial but often underconsidered. The need to address information access inequalities was identified as a major challenge.

Although performance evaluation is not often current practice, most respondents (82%) agreed that using Knowledge, Attitude, Practice and Behaviour surveys to evaluate campaigns would be beneficial. Two thirds (68%) agreed that specific evaluation methods should be integrated, while 50% supported using general NCD surveys like WHO STEPwise. Other methods, such as monitoring the uptake of NCD prevention services (for example, screening, risk assessment, dietary advice or ending tobacco use) were mentioned by 15% of respondents.

**Fig. 2:**
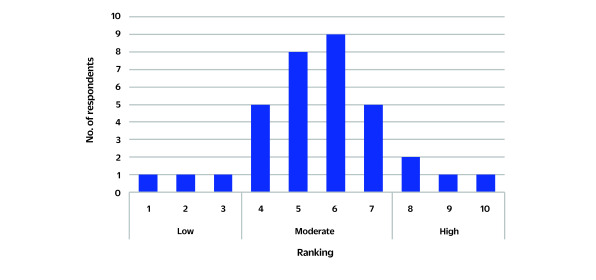
Distribution of survey respondents' success ranking of NCD communication campaigns (*N* = 34), August–October 2023

Most respondents rated their campaigns as moderately successful (**Fig. 2**). Responses to open-ended questions providing qualitative information on respondents’ perception of success factors and challenges are summarized in **Table 1**, grouped by theme (execution, relevance and engagement). Respondents highlighted that campaign execution success was enhanced by access to adequate funding, trained staff, effective planning, use of evidence-based messaging, consistent strategies (for example, annual campaigns), robust evaluation tools (for example, pre-post measures, behavioural outcomes) and policy support (for example, tobacco taxes). Challenges included limited funding and resources, staffing shortages, geographical barriers, inconsistent leadership and low-quality campaign evaluations. Respondents stressed the importance of approaching NCD communication collaboratively by involving multiple sectors, engaging more with civil society and local/traditional structures, and adopting a consistent approach. Respondents mentioned the need to invest more resources in planning and evaluation, including implementing a testing phase, to understand more precisely what makes the message “work” and “focusing more on how to do this.” In relation to planning, respondents also spoke of the need to be “ready for a high level of public response and ensuring that the infrastructure can handle this, especially support or screening services.”

[**insert** Figure 2]

Messaging relevance was considered most successful when tailored, relatable and actionable messages were targeted at clearly defined audiences, delivered through multiple channels and repeated consistently. Current challenges arose from inadequate audience research, low community involvement and reliance on non-local materials that lacked cultural relevance.

Respondents agreed that audience engagement is key for impact but said that it was not practiced enough. They stressed the importance of managing public perceptions of health issues, particularly when sources of information abound and when interpretation can differ between communicators and audiences. They recommended collaborating with multiple stakeholders, forming cross-sector partnerships, involving target audiences in message design, and providing accessible follow-up services. They highlighted the catalytic role played by laws and policies (when enforced) to facilitate behaviour change.

Common barriers included tackling what were referred to as “public misconceptions” around health (for example, “obesity not necessarily perceived as an issue”), difficulty engaging specific audiences, such as youth and generic campaigns that overlook community needs, the inability of the public to initiate and sustain behaviours due to their living or social environments (for example, “high cost and seasonality of healthy food options”), and competition from well funded unhealthy product advertising within the same realm of communication. Respondents recommended collaborating with  “well-known individuals in communities” and “leveraging social networks” to overcome such barriers, as well as using more innovative and “daring” approaches to “keep people motivated,” including one-to-one and group interactions, both in person and through technology and incentives. Continuous support was considered critical to ensuring long-term engagement and helping individuals “stick to behaviours.” Respondents also stressed the need for sustained action within communities, citing the integration of routine health activities into workplaces as a case in point.

### Poster analysis

We analysed 284 public health posters addressing a broad range of health issues including reproductive health, hygiene, parasitic infections, and both communicable diseases and NCDs. NCD-related themes accounted for 54% of all posters. Among the NCD-related posters, themes most depicted were diet and nutrition (63%), tobacco (10%) and diabetes (8%).

Most posters featured cultural references specific to the Pacific or individual PICTs, incorporating local characters, traditional patterns and typical landscapes. Approximately one third used vernacular languages, and another third used English. Bilingual and trilingual posters were less common, but the latter were more common than the former due to contributions from Vanuatu and Wallis and Futuna.

Posters primarily adopted authoritative injunctions (46%) or neutral tones (44%), with fewer using enthusiastic, humorous or dramatic tones. Most posters did not include explicit motivators, but when motivators were present, positive motivators were most prevalent: “happiness” was the most common (22–23%), followed by “fear” (11%). The majority of NCD posters were not targeted at specific subpopulations (73%). Specific age groups were targeted in 14% of posters, genders in 10% and ethnic groups in 8%.

**Fig. 3:**
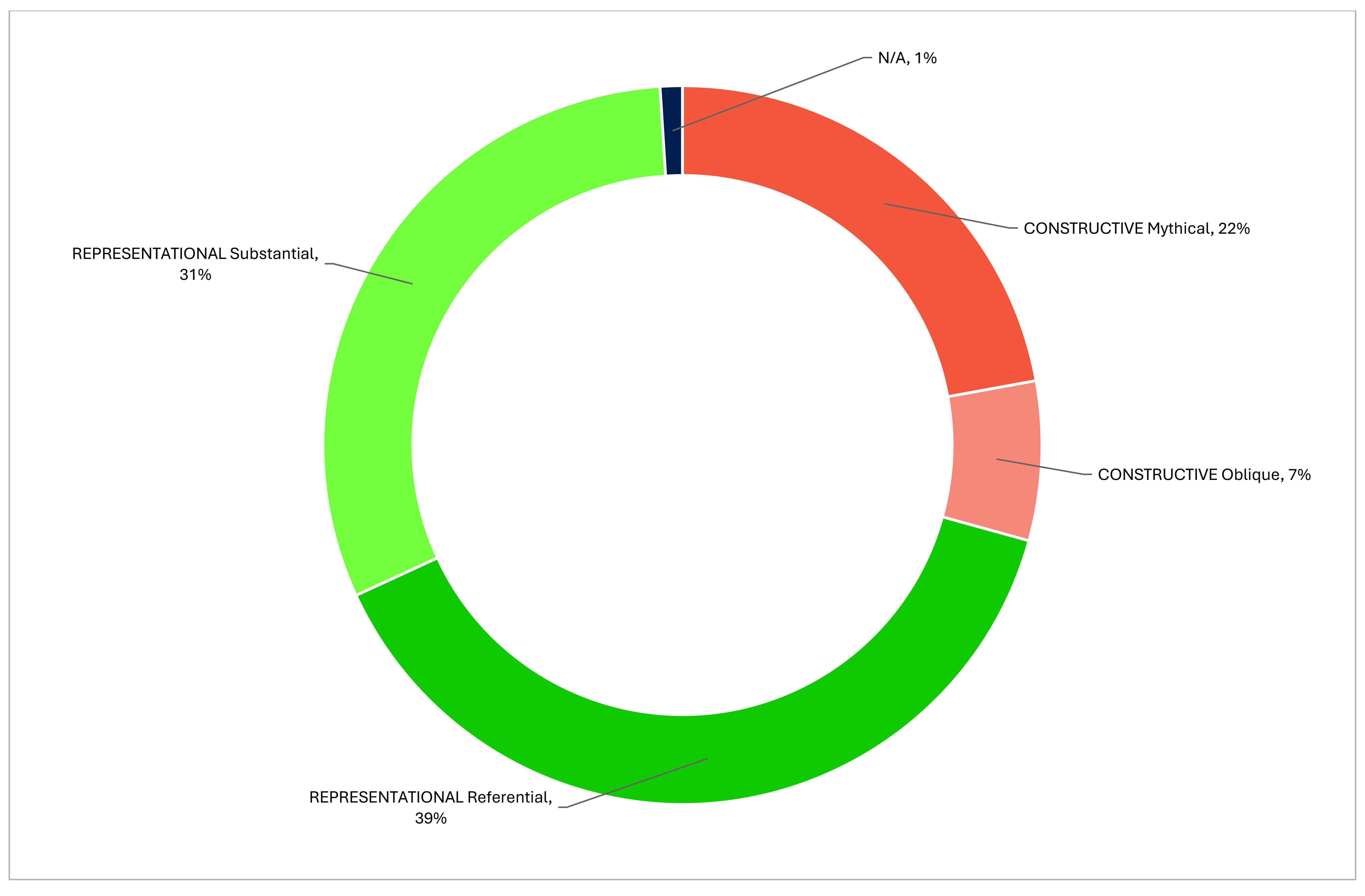
Distribution of postersa by function (*N* = 153)^a^

The majority of NCD posters (70%) served the representational function, portraying reality as designed by public health authorities (**Fig. 3**). Slightly more than half of the representational NCD posters were referential (providing factual content), and just under half were substantial (using aesthetics to evoke emotions). Most of the remaining posters (29%) were constructive, but within this subset, most were “mythical” posters relying on cultural references to associate health with positive values (**Fig. 1**). NCD posters predominantly used artistic drawings (50–56%) and photographs (27–37%). The choice of style often reflected the intended function, with artistic drawings used for constructive posters and photographs for representational ones.

[**insert** Figure 3]

## Discussion

Communication campaigns are widely used to raise awareness of preventable diseases and promote healthier behaviours. However, their impact – as most of the respondents in this study acknowledged – is often mixed, in part due to underlying social and structural conditions that shape health-related behaviours, but are less amenable to change. (*12*)

A central challenge is that messaging alone rarely achieves behaviour change. (*13*) While cognitive or attitudinal shifts may occur, their translation into lasting behaviours is difficult, especially for NCD risk factors because recommended behaviours often conflict with ingrained, highly socialized habits and require immediate costs without immediate rewards. (*14*) Nevertheless, there have been some successes in the region. Studies have shown that campaigns can influence social norms when they are rigorously planned and use graphic, emotionally resonant messages and multiple platforms. (*15*) Anti-smoking campaigns exemplify this. Emotive and evidence-based messaging sustained over time, coupled with legislation and regulation enforcement, can cut across demographics and be effective. As a result, tobacco use has been steadily declining across the region. (*16*)

Our survey responses reflected a strong belief in the importance of context and the need for messaging to be accompanied by broader measures, such as healthier food access, fiscal incentives, and infrastructure and regulatory measures. Several respondents mentioned the crucial roles played by workplaces, communities, laws and policies (particularly in the case of tobacco) in sustaining change. While support for a settings approach that addresses “people and places” (*13*) is strong among PICTs, (*17*) implementation of supportive measures and policies is often limited in practice (*7*) and hindered by technical and political barriers. (*12*, *18*, *19*) This perspective was echoed by our respondents who listed suboptimal support from leaders and legislators as a key barrier to successful communication campaigns, along with poor planning, ill-defined communication strategies, limited resources and weak interdisciplinary collaboration.

Consistent with reports in the literature, our respondents noted that few communication campaigns are adequately informed by preliminary research, leading to critical social, cultural, economic and political dynamics being largely ignored. As a result, campaigns tended to overemphasize personal responsibility while neglecting systemic factors. (*19*-*21*) Moreover, as several respondents noted, communication campaigns for NCD prevention can easily be overshadowed by the commercial marketing of unhealthy products, which often leverage these social and cultural dynamics more effectively. (*11*)

Other shortcomings in NCD prevention communication highlighted by our respondents included insufficient cultural competency and insufficient local adaptation. They mentioned, for example, that local concepts of health and traditional medicine are generally not considered and that messages are often “imported from developed countries.” Studies have shown that most tailored interventions in the Pacific lacked cultural competency and sustainability. (*22*) This is exemplified by the case of obesity which, as indicated by respondents, is considered a sign of high social status and thus desirable in many Pacific island societies. Anthropologists have pointed out that obesity in the region has also arisen from complex societal interdependencies that are not addressed let alone understood by standardized media campaigns. (*23*, *24*) In contrast, there is ample evidence that culturally adapted approaches rooted in local traditions and values are more effective and can improve clinical outcomes. (*22*, *25*, *26*) It has also been suggested that Pacific communities’ interconnectedness, which in some ways contributes to NCD risk, could also be harnessed to do the opposite, that is, promote healthy behaviours. (*20*, *24*)

Perhaps not surprisingly, and as noted by our respondents, digital media are increasingly the favoured communication channels due to their low cost, wide reach and interactivity. The evidence relating to social media’s effectiveness is mixed. Some studies showed minimal impact, (*27*) while others found that in a hyperconnected world, peer influence and community reinforcement can facilitate positive change. (*21*, *28*) However, respondents warned of growing inequalities in information access and the new challenges posed by social media health communication, concerns also expressed in the literature. The 2019 measles crisis in Samoa (*29*) and the COVID-19 pandemic are just two examples of how misinformation and “deliberate obfuscation” can influence opinion and policy-making in public health. (*30*)

Several respondents highlighted the importance of community engagement as a driver of successful communication. Numerous Pacific scholars have advocated for evidence-based, community-driven efforts to enhance cultural specificity of health promotion messaging and ensure local ownership. (*20*, *26*, *31*) Yet in practice, and as noted by our survey respondents, community involvement in framing messages is often limited due to resource constraints. Respondents also linked poor community engagement to insufficient contextualization of messaging. Health promotion scholars agree (*32*) and some sociologists argue that NCD prevention messaging has tended to over-rely on individual responsibility, holding individuals “morally accountable” for disease prevention and public health. This has obscured the structural determinants of disease and thus discounted the need for more fundamental change. (*18*) This past focus on the individual was also evident from the poster analysis.

Theory-driven approaches to intervention design are emerging as a potentially effective way of overcoming recognized health communication challenges. The COM-B model and Behaviour Change Wheel, developed by behavioural epidemiologists and psychologists, offer practical tools to identify behavioural drivers and link them to effective interventions and supporting policies. Such tools have demonstrated potential to improve the design, implementation and consistency of behaviour change strategies across public health domains. (*33*) Evidence in support of social marketing and “engaging communication” as alternatives to traditional persuasive strategies is also growing. (*34*-*37*) Social marketing employs segmentation, competition analysis, positive messaging and audience involvement. Successful interventions like TRUTH and VERB use counter-marketing to challenge harmful products, (*38*) and adaptations for Pacific islander communities in New Zealand have shown positive outcomes among youth. (*39*) While a systematic review (*40*) concluded that even partial use of social marketing elements can yield more positive results than conventional campaigns, social marketing is no panacea. Its full potential remains unrealized in the Pacific, where the complete social marketing mix – product, price, place, promotion – is rarely applied in the public health field. (*15*)

We acknowledge several limitations in our study. The online survey was conducted using a relatively small number of public health practitioners, which may have affected the generalizability of our findings. The use of a non-standardized questionnaire limited comparability with other studies. The reliance on self-reported data represents another potential source of bias, including the possibility of socially desirable responses. Similarly, the degree of subjectivity in our analysis of public health posters, which was based on semiotic interpretation, did not include data on audience reception. This limited our ability to assess how messages were perceived by target populations. Moreover, while posters are a widely used, longstanding and common communication tool across the region, they represent only one medium among a broader array of strategies. Despite these limitations, we believe our findings remain relevant and meaningful within the scope of our study and may be considered by health authorities in the region seeking to strengthen their NCD prevention communication endeavours.

### Conclusion

NCD communication campaigns in the Pacific have heightened awareness and broadened public dialogues about health. However, they appear to have often fallen short in prompting sustained behavioural change. Our study suggests that to enhance their impact, campaigns must evolve beyond top-down, information-driven tactics and be integrated into broader policy measures and structural reforms. Our findings are consistent with both the current literature and the paradigm that effective communication for NCD prevention should be part of a more systemic approach that addresses living conditions, market trends, cultural nuances and community dynamics, while leveraging interconnectedness and new technologies.

Future efforts should aim to transform the interplay between individuals, their environment and the commercial influences that impact NCD risk vulnerability. The focus should be on four priorities throughout the communication cycle, from design to evaluation: formative research; the systematic evaluation of current health communication practice; cultural tailoring; and community engagement.
